# Microbiological characterization of post-eruption “snowblower” vents at Axial Seamount, Juan de Fuca Ridge

**DOI:** 10.3389/fmicb.2013.00153

**Published:** 2013-06-17

**Authors:** Julie L. Meyer, Nancy H. Akerman, Giora Proskurowski, Julie A. Huber

**Affiliations:** ^1^Josephine Bay Paul Center, Marine Biological LaboratoryWoods Hole, MA, USA; ^2^School of Oceanography, University of WashingtonSeattle, WA, USA

**Keywords:** hydrothermal vents, *Epsilonproteobacteria*, snowblowers, eruption, subseafloor

## Abstract

Microbial processes within the subseafloor can be examined during the ephemeral and uncommonly observed phenomena known as snowblower venting. Snowblowers are characterized by the large quantity of white floc that is expelled from the seafloor following mid-ocean ridge eruptions. During these eruptions, rapidly cooling lava entrains seawater and hydrothermal fluids enriched in geochemical reactants, creating a natural bioreactor that supports a subseafloor microbial “bloom.” Previous studies hypothesized that the eruption-associated floc was made by sulfide-oxidizing bacteria; however, the microbes involved were never identified. Here we present the first molecular analysis combined with microscopy of microbial communities in snowblower vents from samples collected shortly after the 2011 eruption at Axial Seamount, an active volcano on the Juan de Fuca Ridge. We obtained fluid samples and white flocculent material from active snowblower vents as well as orange flocculent material found on top of newly formed lava flows. Both flocculent types revealed diverse cell types and particulates when examined by phase contrast and scanning electron microscopy (SEM). Distinct archaeal and bacterial communities were detected in each sample type through Illumina tag sequencing of 16S rRNA genes and through sequencing of the sulfide oxidation gene, *soxB*. In fluids and white floc, the dominant bacteria were sulfur-oxidizing *Epsilonproteobacteria* and the dominant archaea were thermophilic *Methanococcales*. In contrast, the dominant organisms in the orange floc were *Gammaproteobacteria* and *Thaumarchaeota* Marine Group I. In all samples, bacteria greatly outnumbered archaea. The presence of anaerobic methanogens and microaerobic *Epsilonproteobacteri*a in snowblower communities provides evidence that these blooms are seeded by subseafloor microbes, rather than from microbes in bottom seawater. These eruptive events thus provide a unique opportunity to observe subseafloor microbial communities.

## Introduction

Most of the volcanic activity on Earth occurs on the seafloor and eruptions at mid-ocean ridges may have profound impacts on global biogeochemical cycles (Baker et al., 2012). During submarine eruptive events, intense changes in the circulation of subseafloor fluids flush both fluids and microbes from within the crust out into the water column, including extremely high concentrations of dissolved gases such as CO_2_ and H_2_S that fuel chemolithoautotrophic communities (Delaney, 1998). One remarkable consequence of these changes is the ephemeral phenomenon known as a snowblower vent, first observed at 9°N on the East Pacific Rise in 1991 (Haymon et al., 1993). Haymon et al. found widespread diffuse flow following an eruption, including new venting sites that they termed “snowblower vents” because of the abundant white flocculent material emanating from the seafloor. While the microbial populations creating this bloom were not identified, it was postulated that the white floc was created by sulfide-oxidizing bacteria taking advantage of an increase in available hydrogen sulfide. The white floc producers were thought to be members of the *Epsilonproteobacterial* genus *Arcobacter* since an isolate of this genus from salt marshes was shown to create similar white flocculent material composed of excreted elemental sulfur in a lab-based bioreactor (Taylor and Wirsen, 1997; Wirsen et al., 2002). Rapid production of white floc was later also observed using *in situ* colonization experiments and in shipboard bioreactors inoculated with filamentous white mat collected at non-eruptive diffuse flow vents at 9°N (Taylor et al., 1999). The white floc producers in both the colonization traps and the shipboard bioreactors were later identified as two different *Arcobacter* groups (Sievert et al., 2008a). Despite the evidence that *Arcobacter* from multiple habitats can produce white floc, there has been no direct evidence that *Arcobacter* are present or produce the white floc in snowblower vents.

In addition to active snowblower vents, orange flocculent material was observed coating the seafloor surrounding diffuse flow sites following multiple seafloor eruptions. Orange floc collected following the 1993 eruption at the CoAxial Segment of the Juan de Fuca Ridge consisted of diverse aggregates of cells that were coated with iron and silica, many of which did not stain with DAPI (Juniper et al., 1995). Carbon fixation through RuBisCo activity and the oxidation of hydrogen sulfide were detected in this orange floc, indicating an active microbial community. In addition, thermophiles and hyperthermophiles were detected by enrichment culture from low-temperature diffuse fluids sampled for several years following the 1993 CoAxial eruption (Holden et al., 1998). Some of these enrichment cultures were capable of producing white flocculent material similar in appearance to the floc emanating from snowblower vents (Holden et al., 1998). The production of floc in cultures from both filamentous white mats and diffuse fluids collected between eruptions suggests that the floc producers responsible for snowblowers are long-term residents of vent habitats that bloom during the surge of geochemical fuels concurrent with eruptions. Taken together, these early examinations of eruptive materials suggest that the flocculent material characterizing snowblower vents is generated by a bloom of sulfide-oxidizing bacteria producing elemental sulfur that may become coated with iron and silica as the bloom ages.

Axial Seamount is an active submarine volcano along the Juan de Fuca Ridge that has been closely monitored for more than a decade and is now part of the networked seafloor observatory being installed as part of the Regional Scale Nodes component of the NSF's Ocean Observatories Initiative. Axial Seamount erupted in 1998 and several post-eruption time series studies were performed to monitor changes in the chemistry and microbiological communities in diffuse fluids from the Marker 33 vent (Huber et al., 2003, 2002; Butterfield et al., 2004). Although snowblower vents were observed following the 1998 eruption, the microbial populations were not sampled from these short-lived diffuse flow sites (Butterfield et al., 2004; Chadwick et al., 2013). Fluids from Marker 33 were very gas-rich in the first year after the 1998 eruption and contained high levels of H_2_S and CO_2_, making sulfide oxidation the dominant source of chemical energy for microbial metabolisms (Butterfield et al., 2004). While the energy available from methanogenesis was much lower than that from sulfide oxidation (Butterfield et al., 2004), both putatively mesophilic and hyperthermophilic methanogens were detected in the three years following the eruption (Huber et al., 2002). Temporal changes in the archaeal communities at Marker 33 corresponded with the changing chemistry of the fluids, including an increase in thermophilic *Methanococcus* and a decrease in the *Thaumarchaeota* Marine Group I common in bottom seawater as fluid temperatures increased (Huber et al., 2002). In a separate study of the bacterial community response following the eruption, lower diffuse fluid temperatures and the corresponding increase in oxygen availability favored *Epsilonproteobacteria*, with an increase in the diversity of *Epsilonproteobacteria* over time (Huber et al., 2003).

Axial Seamount erupted again in April 2011 (Caress et al., 2012; Chadwick et al., 2012; Dziak et al., 2012). Fortuitously scheduled research cruises in July and August of 2011 allowed the sampling of both white and orange flocculent types as well as diffuse fluids from active snowblower vents. Here we present the first direct molecular characterization of microbial communities associated with short-lived snowblower vents to examine the major microbial players in orange and white flocs as well as snowblower diffuse fluids. We address how these ephemeral communities relate to more stable diffuse flow vents and the potential sources of the taxa associated with snowblowers.

## Materials and methods

### Site description and sample collection

**Figure 1:**
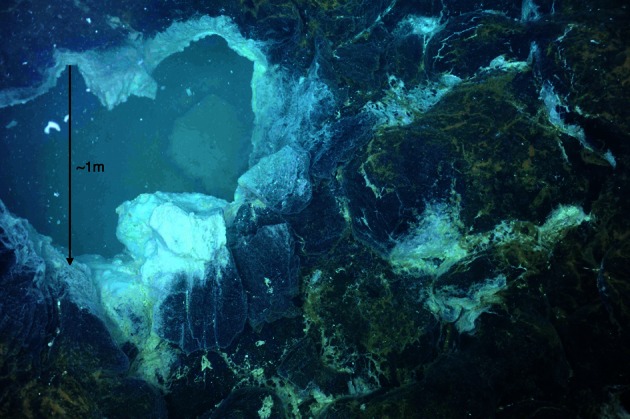
**Seafloor photograph of the “Subway” snowblower vent, Axial Seamount, Juan de Fuca Ridge, showing white flocculent “snow” inside the venting orifice and orange floc coating the surrounding seafloor**. White floc samples 1 and 2 were collected here, while orange floc was collected further away from the venting site.

The April 2011 eruption at Axial Seamount was discovered during a regularly scheduled research expedition in July when seafloor monitoring sites were found buried (Chadwick et al., 2012). Diffuse fluids were collected from newly discovered snowblower vents at Axial Seamount in late July 2011 with the ROV *Jason II* using the hydrothermal fluid and particle sampler (Butterfield et al., 2004). Cells were filtered on the seafloor onto a 0.22 μm Sterivex-GP filter, fixed at 4°C for 24 h with RNA Later immediately upon recovery, then frozen at −80°C until DNA extraction. Whole fluids were fixed with 3.7% formaldehyde for cell counts as previously described (Huber et al., 2002). Basic chemistry for the Snow Globe and Boca snowblower vent fluids was provided by D. Butterfield (personal communication). White and orange flocculent materials were collected on the subsequent University of Washington Visions'11 cruise, in support of the Regional Scale Nodes component of the Ocean Observatories Initiative in August 2011. White flocculent material was collected from the orifice of the Subway snowblower vent (Figure 1) on dives R1467 (White Floc 1) and R1472 (White Floc 2) and orange flocculent material was collected on the seafloor distal to Marker 33 during dive R1472 where it coated freshly deposited basalt (Video S1). All of the fluid and floc samples analyzed in this study are from a small area in the south rift zone at the southeastern edge of Axial Caldera, with the exception of background seawater which was collected outside of the caldera (Table 1).

**Table 1:** **Sampling site descriptions**.

**Sample**	**Vent**	**Latitude/Longitude**	**Depth (m)**	***T*max (°C)**	***T*avg (°C)**	**pH**	Cells g^−1^ of wet floc ^*^ or cells ml^−1^ of fluid ^**^ (± 95% confidence level)
**SOLIDS**							
White Floc 1	Subway	45.942/−129.985	1517	ND	ND	ND	8.6 × 10^8^ (± 2.7 × 10^7^)^*^
White Floc 2	Subway	45.942/−129.985	1516	ND	ND	ND	9.8 × 10^8^ (± 2.0 × 10^7^)^*^
Orange Floc	Marker 33	45.933/−129.982	1515	ND	ND	ND	3.4 × 10^7^ (± 1.2 × 10^6^)^*^
**FLUIDS**							
FS825	Snow globe	45.946/−129.985	1524	12	11.4	5.5	3.0 × 10^5^ (± 1.5 × 10^4^)^**^
FS834	Boca	45.928/−129.985	1519	17.1	16.8	5.4	3.3 × 10^5^ (± 1.1 × 10^4^)^**^
Seawater	−	45.947/−129.984	1526	2.5	2.4	7.8	2.8 × 10^4^ (± 9.6 × 10^2^)^**^

ND, not determined.

### Microscopy

Subsamples of flocculent material were viewed under phase contrast, fixed and stained with DAPI for cell counting via epiflourescent microscopy, or fixed for scanning electron microscopy (SEM) with elemental detection system (EDS) on a Zeiss Supra 40VP. White and orange flocs were fixed with 2.5% glutaldehyde for both cell counts and SEM. Samples were prepared for SEM by dehydration in a series of chilled alcohol washes (50, 70, 85, 95, 100%), dried with a critical point dryer, mounted, and sputter-coated with platinum. Preserved whole fluids were also stained with DAPI for cell counts.

### Bulk carbon, nitrogen, and sulfur measurements

Bulk carbon and nitrogen were measured using a Thermo Scientific CN Analyzer (Model Flash 2000) from subsamples of flocculent material dried at 50–60°C for 2 days. A standard curve for bulk carbon and nitrogen was made using aspartic acid as a standard and acetanilide and apple leaf as standard curve checks. Bulk sulfur was measured using a LECO S632 Sulfur Analyzer from subsamples of flocculent material dried in a dessicator for 4 days and supplemented with sterile sea sand (Fisher) to meet minimum volume requirements. A standard curve for bulk sulfur was made using coal with known sulfur content provided by LECO. Two subsamples were analyzed for each floc sample and replicate readings were averaged for all bulk C, N, and S measurements.

### DNA extraction and illumina tag sequencing

Total genomic DNA was extracted from Sterivex filters as previously described (Sogin et al., 2006) with the minor modifications described by Akerman et al. (in review). Total genomic DNA was extracted from 20 to 30 mg of wet flocculent material using a MoBio UltraClean® Soil DNA Isolation Kit. The V6 region of 16S rRNA genes were amplified in triplicate for each sample with previously reported primers designed for archaea and bacteria (Huber et al., 2007) that were modified to include indices and barcodes compatible with the Illumina HiSeq1000 platform rather than 454 Life Sciences Adapters (Eren et al., 2013). Triplicate PCR amplifications were pooled for each sample, cleaned with a Qiagen MinElute kit, and quanitified by PicoGreen assay on a Turner Biosystems spectrophotometer. Fifty nanograms of each cleaned amplicon library was then size selected with a 2% agarose PippinPrep cassette to produce a narrow range of fragment sizes from 200 to 300 bp for sequencing and cleaned again to remove agarose. All of the amplicon libraries included in this study were sequenced in the same run and on the same paired-end lane, along with 60 other libraries. Equimolar amounts of pooled amplicon libraries and a metagenomic library were run in the same lane to avoid known difficulties of sequencing low-complexity amplicon libraries with Illumina (Caporaso et al., 2012).

### Sequence analysis

Paired Illumina sequencing reads were quality filtered to remove any reads containing ambiguous nucleotides and only pairs with perfectly overlapping reads were used for further analysis. Quality-filtered reads are publicly available through the VAMPS database, http://vamps.mbl.edu under the project name JAH_AXV_Bv6 and JAH_AXV_Av6, where orange floc is listed as “eruption mat,” white floc 1 is listed as “snow_R1467,” and white floc 2 is listed as “snow_R1472.” Sequences were clustered at 97% similarity with a minimum word length of 30, using usearch (Edgar, 2010). Taxonomy was assigned by global alignment for sequence taxonomy (GAST; Huse et al., 2008) with the SILVA 111 database (Quast et al., 2012). Operational taxonomic units (OTUs) were then analyzed with Qiime 1.5 (Caporaso et al., 2010). Even sequencing depth per sample was established by multiple rarefactions to roughly 75% of the smallest sequencing depth, using a total of 195,000 bacterial reads and 145,000 archaeal reads per sample. To compare bacterial communities in snowblower fluids and flocculent samples to background seawater by dendrogram, we retrieved bacterial V6 454 reads from background seawater collected outside the Axial Caldera from the VAMPS database under the project name KCK_SMT_Bv6, fluid sample FS501. To compensate for the fewer number of reads in the background seawater sample, a second set of multiple rarefactions was performed with 7112 reads per sample. Distance matrices were calculated for 10 rarefactions using the Morisita-Horn index (Horn, 1966) and the resulting tree topographies were clustered using UPGMA to create a final jackknifed tree.

### Sulfur oxidation genes

The oxidation of reduced sulfur compounds, including hydrogen sulfide, thiosulfate, elemental sulfur, and sulfite, can be achieved through the Sox pathway, which is present in several genera of *Epsilonproteobacteria* in hydrothermal vent systems (Yamamoto and Takai, 2011). As the sulfur-oxidizing genera *Sulfurovum* and *Sulfurimonas* are often abundant in 16S rRNA gene libraries from Axial Seamount (Huber et al., 2007), we targeted the amplification of the *soxB* gene in *Epsilonproteobacteria* to assess the diversity of sulfur oxidizers in white floc vs. orange floc samples. The *soxB* gene was amplified in one white flocculent sample (White Floc 2) and in the orange flocculent sample, using the newly designed primers and conditions described by Akerman et al. (in review). The PCR reaction mixture consisted of 1X buffer (Promega), 4 mM MgCl_2_, 0.2 mM of each deoxynucleoside triphosphate (dNTP), 0.6 μM of each primer, 1 U GoTaq polymerase (Promega), 1 μl DNA template, and DEPC H_2_O to 25 μl. Thermocycling conditions on an Eppendorf thermal cycler consisted of an initial denaturation step at 94°C for 3 min, followed by 35 cycles of 94°C for 30 s, 46°C for 45 s, and 72°C for 1 min, followed by a final extension at 72°C for 5 min. The primers used in this study were sox527F (5′-TGGTWGGWCAYTGGGAATTTA-3′) and sox1198R (5′-AGAANGTATCTCKYTTATAAAG-3′). These primers target the genera *Sulfurovum, Sulfurimonas*, and *Nitratiruptor*. The *soxB* gene in members of the *Epsilonproteobacterial* genera *Arcobacter* and *Nitratifractor* are more like sequences in *Gammaproteobacteria* and are not expected to amplify with this primer set. Successfully amplified *soxB* PCR products were cleaned with a Qiagen MinElute PCR purification kit and run on a 0.8% agarose gel. Bands in the expected size range were gel excised, purified with the MinElute kit, cloned, and sequenced as previously described (Huber et al., 2009). Nucleotide sequences were translated into amino acids using EMBOSS Transeq (Rice et al., 2000) and phylogenetic relationships were analyzed using MEGA5 (Tamura et al., 2011). Sequences are deposited in GenBank under Accession numbers KC793341-KC793425.

### Quantitative PCR (qPCR)

The relative abundances of bacteria and archaea were determined by qPCR TaqMan assays as previously described (Huber et al., 2010). Briefly, standards were constructed from linearized plasmids of Axial diffuse vent clone libraries. Each 20 μl reaction contained TaqMan Gene Expression Master Mix (Applied Biosystems), forward and reverse primers of 9 μM (bacteria) or 8 μM (archaea), probe concentrations of 1.5 μM (bacteria) or 2 μM (archaea), DEPC-treated water, and 2 μl of DNA template. Triplicate reactions were performed on a StepOne Plus Real Time PCR System (Applied Biosystems) for each sample and for no template controls. Standard curves had *R*^2^ values > 0.997 and efficiencies ranging from 89 to 96%.

## Results

### Microscopy

**Figure 2:**
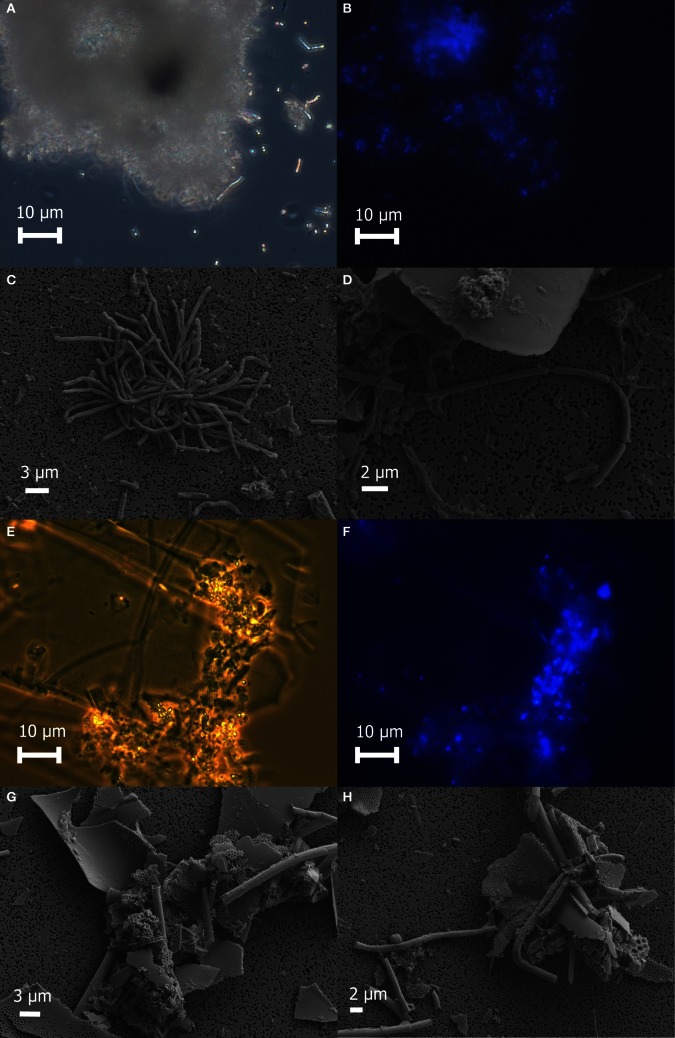
**Microscopic examination of white floc 2 (panels A through D) and orange floc (panels E through H)**. Paired phase contrast **(A)** and epiflourescent **(B)** images of DAPI-stained cells in white flocculent material from the Subway snowblower vent. SEM images of white floc show large clumps **(C)** and filaments **(D)** of microbial cells. Paired phase contrast **(E)** and epiflourescent **(F)** images of DAPI-stained cells in orange flocculent material from the seafloor surrounding Marker 33. SEM images of orange floc showing eukaryotic debris and hollow sheaths **(G** and **H)**.

Freshly prepared slurries of white flocculent material contained very fine particulates, giving an overall milky appearance. Examination of this white floc under phase contrast revealed copious amounts of small bright spheres around 1 μm in diameter and rods up to 20 μm in length, presumably made up of or coated with elemental sulfur, as well as single rods or large clumps of cells and debris (Figure 2A). None of the bright spheres or rods could be stained with DAPI (Figure 2B) and were not identified by SEM (Figures 2C,D), likely because the elemental sulfur was removed during the dehydration alcohol washes. Only small quantities of sulfur were detected by EDS. Large clumps of filamentous cells as well as debris from eukaryotic cells were visible by SEM (Figures 2C,D). Sheet-like structures and other eukaryotic debris were mostly made up of silica and oxygen, with traces of iron, calcium, magnesium, and aluminum. Nitrogen and potassium were detected only in the large clumps of filamentous cells.

Fresh slurries of the orange floc contained much larger particulates that were visible to the naked eye and readily settled out of solution. Examination of orange floc under phase contrast showed that this floc is composed mostly of the same kind of eukaryotic debris in the white floc as well as likely iron oxides, but generally lacking in the bright spheres and rods seen in the white floc (Figures 2E–H). Aggregates of debris contained microbial cells that stained with DAPI (Figures 2E,F). Sheath-like structures were visible by both phase contrast and SEM in the orange floc (Figures 2G,H) but not in white floc.

Cell counts revealed higher numbers of cells in the white floc than in orange floc and higher numbers of cells in the vent fluids compared to background seawater (Table 1).

### Characterization of floc and fluids

Bulk carbon, nitrogen, and sulfur values were obtained for both white and orange flocculent materials (Table 2). Total organic carbon ranged from 0.73 to 1.82% and nitrogen ranged from 0.16 to 0.45% of dry weight. Total sulfur ranged from 0.79 to 37.9% of dry weight. Both white floc samples contained much higher proportions of sulfur than the orange floc. Fluid chemistry from both Snow Globe and Boca vents had elevated temperatures and lower pH compared to background seawater (Table 1). In addition, dissolved silica in the snowblower fluids was three times higher than in background seawater and hydrogen sulfide concentrations were greater than 100 μM (D. Butterfield, personal communication).

**Table 2:** **Bulk carbon, nitrogen, and sulfur measurements of flocculent material**.

**Sample**	**% Carbon**	**% Nitrogen**	**% Sulfur**
White Floc 1	1.83	0.45	37.9
White Floc 2	0.71	0.16	6.66
Orange Floc	1.28	0.16	0.79

### Community composition

**Figure 3:**
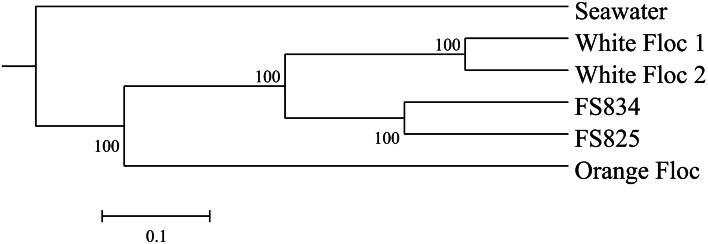
**UPGMA clustering tree of the bacterial community structure**.

Genomic DNA was successfully extracted and amplified from all samples. Over three million perfectly overlapping reads were obtained for bacterial and archaeal amplicon libraries (Table 3). At the 3% clustering level, a total of 8228 OTUs were obtained for bacteria and a total of 2352 OTUs were obtained for archaea. The smaller subset of reads used for the dendrogram with background seawater had a total of 1508 OTUs at the 3% clustering level. The microbial communities clearly clustered by sample type (Figure 3). Using the full set of bacterial and archaeal reads yielded the same topography within the snowblower fluid and flocculent samples (data not shown). White floc and snowblower fluid samples were dominated by *Epsilonproteobacteria*, while orange floc and background seawater were dominated by *Gammaproteobacteria*. All samples were dominated by bacteria, with less than 1% of the community DNA belonging to archaea in the snowblower fluid and floc samples and just under 3% archaea in the background seawater (Table 3).

**Table 3:** **Summary of Illumina sequencing and qPCR results for each sample**.

	**Bacterial V6 quality-filtered reads**	**Bacterial V6 unique sequences**	**Archaeal V6 quality-filtered reads**	**Archaeal V6 unique sequences**	**Bacteria (%)^†^**	**Archaea (%)^†^**
White floc 1	530,098	20,248	314,342	15,281	99.95	0.05
White floc 2	359,002	17,617	216,716	10,914	99.95	0.05
FS825	295,915	20,242	248,244	10,161	99.89	0.11
FS834	265,443	20,243	252,980	10,382	99.66	0.34
Orange Floc	605,312	41,684	196,620	9,781	99.10	0.90
Seawater	−	−	−	−	97.15	2.85
Total	2,055,770	120,034	1,228,902	56,519		

† Based on qPCR as described in Materials and Methods.

### Epsilonproteobacteria

**Figure 4:**
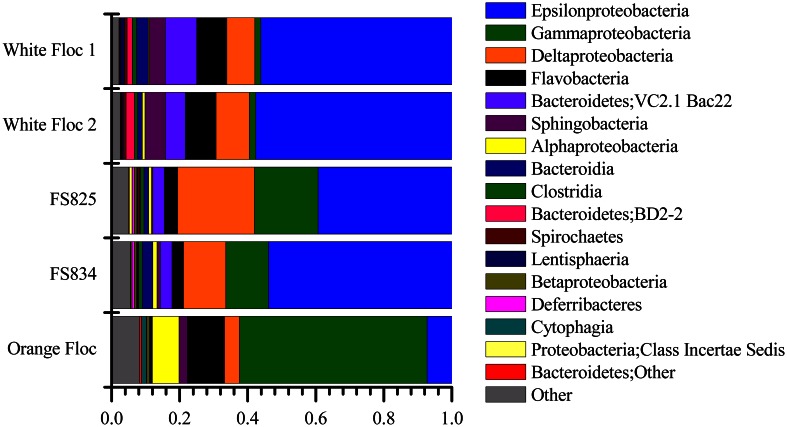
**Relative abundance of dominant bacterial classes in Illumina amplicon libraries from white floc and fluid samples from active snowblower vents as well as orange floc from the surrounding seafloor**.

**Figure 5:**
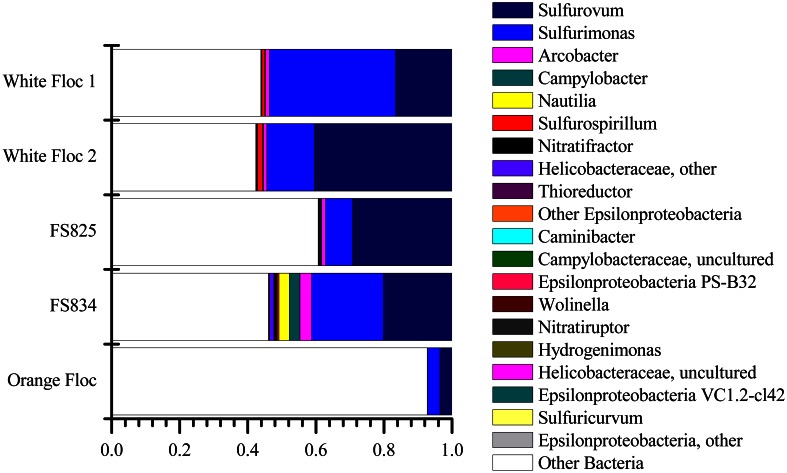
**Relative abundance of *Epsilonproteobacterial* genera in Illumina amplicon libraries from white floc and fluid samples from active snowblower vents as well as orange floc from the surrounding seafloor**.

The *Epsilonproteobacteria* dominated the bacterial communities from snowblower vents (Figure 4). By far, the two most dominant groups of *Epsilonproteobacteria* were *Sulfurovum* and *Sulfurimonas*, although a total of 20 *Epsilon* genera were detected (Figure 5). In white floc samples, up to 40% of reads were *Sulfurovum* and up to 37% of reads were *Sulfurimonas*. The two snowblower fluid samples were also dominated by *Sulfurovum* and *Sulfurimonas*, but one of the fluid samples had somewhat higher levels of the genera *Arcobacter, Campylobacter, Nautilia*, and an unclassified genus of *Helicobacteriaceae*. *Sulfurospirillum* was a minor component of all of the samples, but appeared more abundant in white floc than in other samples. In orange floc, far fewer reads were assigned to *Epsilonproteobacteria*, with *Sulfurovum* and *Sulfurimonas* making up 3.5 and 3.7% of the reads, respectively. The only *Epsilonproteobacteria* detected in the background seawater were *Sulfurimonas* and an unclassified genus of the same family (*Helicobacteraceae*), both of which were constituted less than 0.02% of reads.

### Gammaproteobacteria

**Figure 6:**
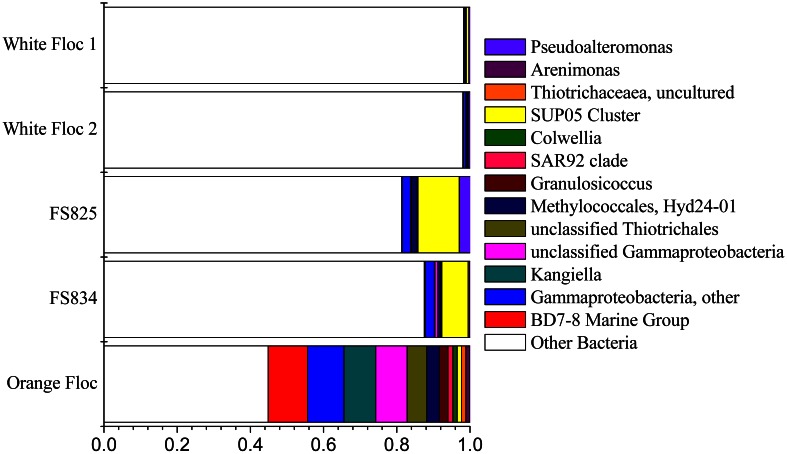
**Relative abundance of *Gammaproteobacterial* genera in Illumina amplicon libraries from white floc and fluid samples from active snowblower vents as well as orange floc from the surrounding seafloor**.

The *Gammaproteobacteria* dominated the orange floc and were also a major component of the snowblower fluid samples (Figure 4). While 55% of the reads from the orange floc and up to 18% of reads in the fluid samples were assigned to *Gammaproteobacteria*, less than two percent of the white floc samples were *Gammaproteobacteria*. Dominant *Gammaproteobacterial* groups in the orange floc included the uncultured deep-sea sediment BD7-8 Marine Group (10.8% of reads), *Kangiella* (8.7%), an unclassified *Gammaproteobacterial* group (8.6%), *Thiotrichales* (5.3%), and *Methylococcales* (3.5%) (Figure 6). In contrast, the dominant group of *Gammaproteobacteria* in the fluid samples was the SUP05 cluster of *Oceanospirillales*, which made up 7–11% of the reads from fluid samples. The SUP05 cluster made up less than 1% of reads in the white floc and just 1.2% of the orange floc.

**Figure 7:**
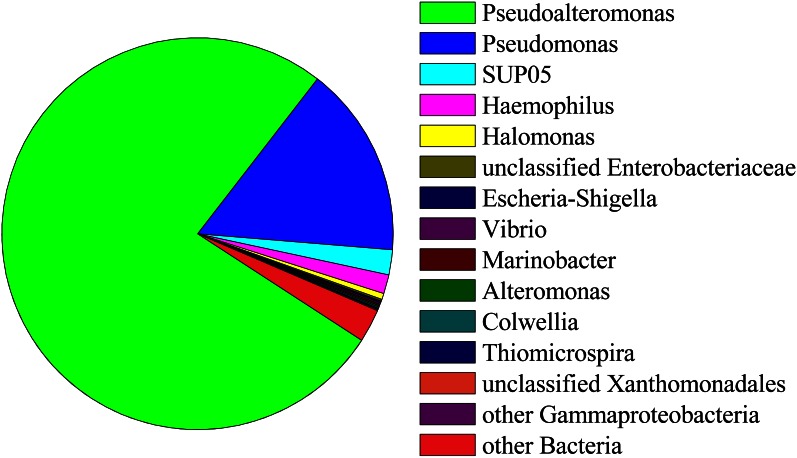
**Relative abundance of 454 reads assigned to *Gammaproteobacteria* in background seawater**.

Ninety-seven percent of the reads in background seawater were *Gammaproteobacteria*, most of which were assigned to the genera *Pseudoalteromonas* (76%) and *Pseudomonas* (16%) (Figure 7). *Pseudoalteromonas* was also present in all snowblower floc and fluid samples, making up 2.9% of the reads in fluid sample FS825, 0.1% of reads in FS834 and the orange floc, and 0.3% of reads in each of the white floc samples. *Pseudomonas* was detected in all of the snowblower floc and fluid samples, but constituted less than 0.14% of reads in any single snowblower sample. SUP05 was also detected in the background seawater, making up about 2% of the reads.

### *Deltaproteobacteria* and *Betaproteobacteria*

*Deltaproteobacteria* were more prominent in the snowblower fluid samples than in other samples (Figure 4). In fluids, the most dominant genera of *Deltaproteobacteria* detected were *Desulfobulbus, Desulfobacterium*, and *Desulfocapsa*, all of which belong to the order *Desulfobacterales*. Several groups of *Bdellovibrionales* were also detected, primarily the OM27 clade and *Peredibacter*. In white floc, the most dominant genera were *Delsulfocapsa* and several genera of *Desulfuromonadales*. In contrast, the most dominant groups in the orange floc were *Myxococcales* and the *Bdellovibrionales* OM27 clade. *Myxococcales* made up 1.6% of reads in the orange floc but only 0.2% of reads in all other samples. *Betaproteobacteria* made up only a minor fraction (<1% of reads) of all fluid and floc samples. The most frequently detected *Betaproteobacterial* genus in each snowblower fluid or floc sample was *Sideroxydans*, which constituted 0.4% of reads in the orange floc, 0.5% of reads in both snowblower fluid samples, and 0.08–0.1% of reads in the white floc. The genus *Leptothrix* was detected only in the orange floc, where this genus was assigned to 0.05% of reads.

### Bacteroidetes

After *Sulfurovum* and *Sulfurimonas*, the third most abundant taxonomic group overall was the uncultured *Bacteroidetes* group defined by the hydrothermal vent clone VC2.1 Bac22 (Reysenbach et al., 2000). This group made up more than 3% of total reads in both fluid samples and in white floc 2 and made up more than 9% of reads in white floc 1. In contrast, less than 0.1% of reads in the orange floc were assigned to *Bacteroidetes* VC2.1 Bac22. Other abundant *Bacteroidetes* groups in fluids and white floc samples included reads that were similar to the *Sphingobacteriales* group WCHB1–69 from a hydrocarbon-contaminated aquifer (Dojka et al., 1998) and an uncultured group of *Marinilabiaceae*. In the orange floc, two uncultured groups of *Flavobacteriaceae* were the dominant *Bacteroidetes*, making up more than 6% of the total reads for orange floc.

### Archaea

**Figure 8:**
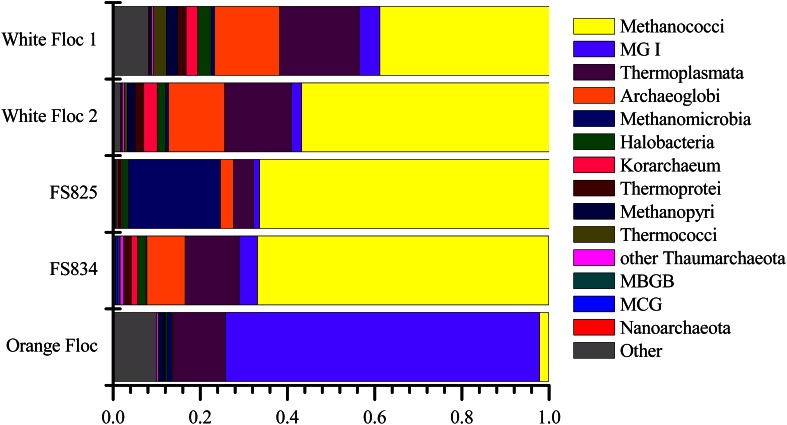
**Relative abundance of dominant archaeal classes in Illumina amplicon libraries from white floc and fluid samples from active snowblower vents as well as orange floc from the surrounding seafloor**.

Although archaea do not make up a large proportion of the microbial communities in these samples, the differences in composition of the archaeal communities in white floc or fluid samples and the orange floc were striking (Figure 8). As in the bacterial community, the archaeal communities in white floc and fluid samples were very similar. The snowblower fluids and white floc were dominated by methanogens, with 42–87% of total archaeal reads assigned to methanogenic groups, while methanogens made up only 4% of archaeal reads in the orange floc. The dominant methanogens in the fluids and white floc were the *Methanococci*, including the genera *Methanococcus, Methanothermococcus*, and *Methanocaldococcus*. The *Methanococcales* made up more than 66% of the reads from fluid samples and 39–57% of the reads from white floc. The *Methanosarcinales* GOM Arc I clade within the *Methanomicrobia* made up almost 20% of the reads in fluid sample FS825 from the Snow Globe vent. Other dominant *Euryarchaeota* groups in the fluid samples and white floc include *Archaeoglobi* and the *Thermoplasmata* clades AMOS1A-4113-D04 and Marine Benthic Group D. In contrast, the orange floc was dominated by *Thaumarchaeota* Marine Group I, which made up more than 71% of the archaeal reads. The orange floc also had reads assigned to the *Thermoplasmata*, but these were representatives of the clades Marine Group II and Marine Group III, which each made up more than 5% of the reads.

### Sulfur oxidation through the sox pathway

**Figure 9:**
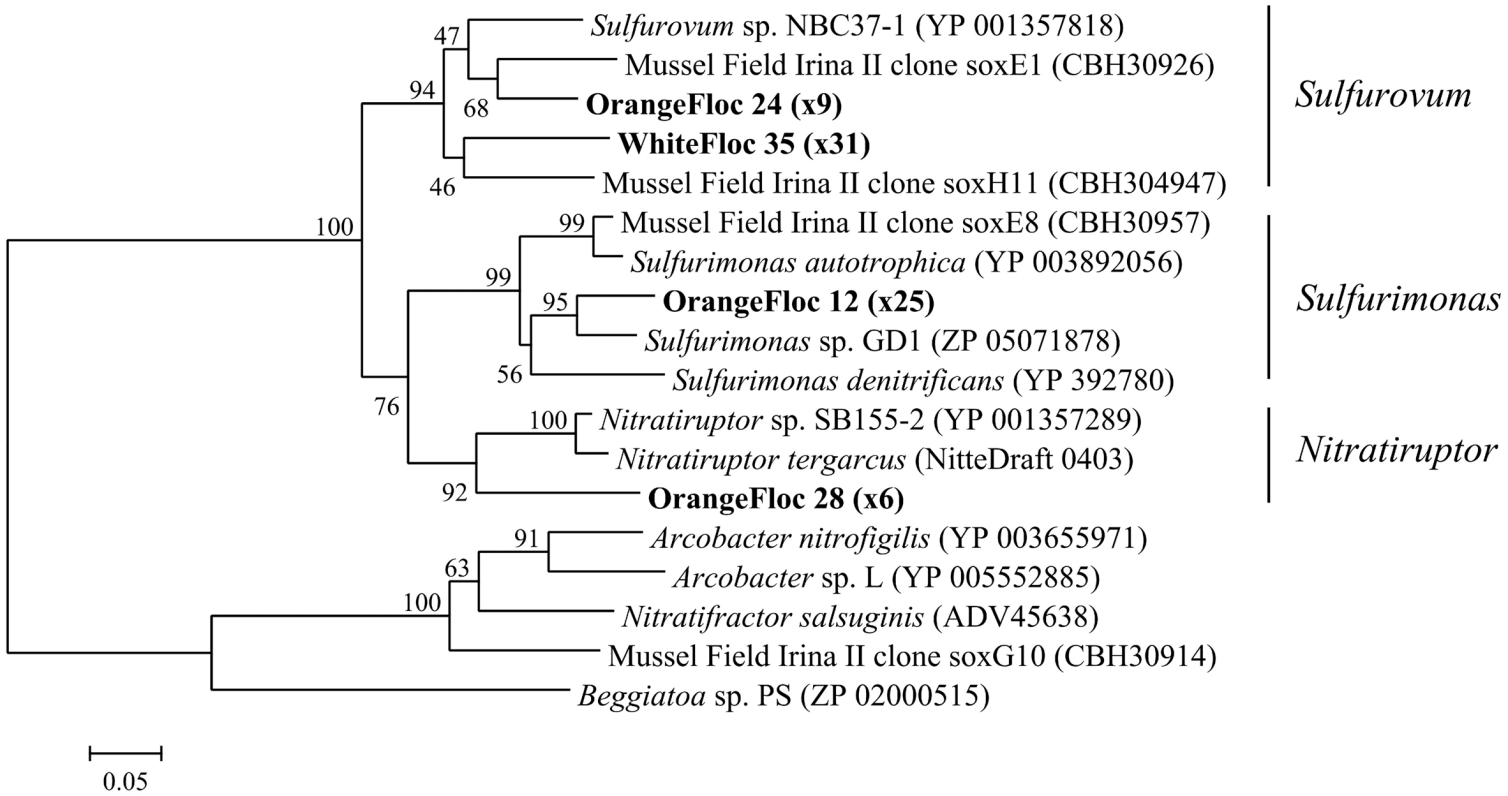
**Neighbor-joining tree of *Epsilonproteobacterial soxB* genes**. The percentage of replicate trees in which the associated taxa clustered together in the bootstrap test of 1000 replicates is shown next to branches. Branch lengths are equal to the evolutionary distance computed using the Poisson correction method and are shown in units of the number of amino acid substitutions per site. Sequences obtained in this study are highlighted in bold. The number of replicate clones is shown in parentheses.

*Epsilonproteobacterial soxB* genes were successfully amplified, cloned, and sequenced from both white floc 2 and orange floc, yielding 31 sequences from white floc 2 and 40 sequences from orange floc. These sequences clustered with *soxB* from *Sulfurovum, Sulfurimonas*, and *Nitratriuptor* (Figure 9). A total of four phylotypes were detected at the 90% amino acid similarity level from these 71 clones, none of which were shared between white and orange floc samples. All clones from the white floc (White Floc 2) were most closely related to *soxB* from *Sulfurovum*, the most dominant genus in this sample, which made up 40% of the 16S rRNA gene sequences. The orange floc also had *Sulfurovum*-like *soxB* genes that were 85% similar to the white floc sequences. In addition, the orange floc had *Sulfurimonas*- and *Nitratiruptor*-like *soxB* sequences. The *Sulfurovum* and *Sulfurimonas soxB* genes detected here were similar to *soxB* genes from the Irina II hydrothermal vent chimney complex at the Logatchev vent field on the Mid-Atlantic Ridge (Hügler et al., 2010) and to *soxB* in the genomes of *Sulfurovum* sp. NBC37-1 (Nakagawa et al., 2007) and several *Sulfurimonas* isolates (Sievert et al., 2008b; Sikorski et al., 2010; Grote et al., 2011; Figure 9).

## Discussion

These results demonstrate that the microbial communities in white floc and fluids from snowblower vents are distinct from those in the orange floc coating fresh basalt following a volcanic eruption. Visual and microscopic evidence suggest the orange floc is made up of larger and heavier particulates than the white floc, especially iron oxides and multi-cellular debris. The orange floc may, in part, be fallout from snowblower vents which becomes colonized by a distinct subset of microbes from the surrounding cold and oxygenated deep seawater, including iron oxidizing bacteria. Given the observed presence of orange floc on both vertical and overhanging surfaces (Chadwick et al., 2013), it is clear that growth of this eruption mat occurs in place on fresh lava flows. While the most dominant bacterial class in both the orange floc and in background seawater was *Gammaproteobacteria*, the most abundant genera detected in each were different. In the orange floc, no more than 10.8% of reads were assigned to any one genus within the *Gammaproteobacteria*, in contrast to background seawater from outside the Axial Caldera, which was dominated almost entirely by just *Pseudoalteromonas* and *Pseudomonas*. The most dominant bacteria in orange floc was the *Gammaproteobacteria* BD7–8 Marine Group which was first identified from deep sea sediments (Li et al., 1999) and has since also been detected in iron rich mats from both Vailulu'u Seamount near American Samoa (Sudek et al., 2009) and Loihi Seamount, near Hawaii (Fleming et al., 2013). While the iron oxidizer *Sideroxydans* was detected at low levels in all snowblower fluid and floc samples, *Leptothrix* was detected only in the orange mat. Both freshwater and marine strains of the iron oxidizing bacteria *Leptothrix* have demonstrated the ability to form sheaths very similar in appearance by phase contrast and SEM (Fleming et al., 2013) to the sheaths we detected only in the orange mat from Axial Seamount. In addition, the orange floc contained higher numbers of reads assigned to *Myxococcales*, a group which has previously been shown as enriched in the particulate fraction of the deep-sea water column (Eloe et al., 2011). Taken together, these results suggest that the orange mat coating freshly deposited lava flows contains iron oxidizing bacteria and other groups which colonize particulates that fall out of eruption-related hydrothermal plumes.

The orange floc still contained low levels of the *Sulfurovum* and *Sulfurimonas* that dominated the bacterial snowblower community. However, it is likely that these sulfide oxidizers are diminishing in the orange floc as the particulates settle out of the snowblower plume and away from the source of sulfide. Bulk sulfur was much lower in the orange floc than in the white floc, indicating that sulfur is not as readily available as an energy source in the orange floc nor is elemental sulfur being copiously produced by sulfur oxidizing microorganisms. The orange floc contained a higher diversity of *Epsilonproteobacterial soxB* genes and these phylotypes were distinct from white floc phylotypes and from the dominant phylotypes in diffuse fluids collected a year prior to the 2011 eruption at Axial Seamount (Akerman et al., in review). The *soxB* phylotypes in orange floc may therefore represent *Epsilonproteobacteria* that are adapted to cooler, more aerobic habitats than the phylotypes found in the white floc and non-eruptive diffuse fluids.

The white floc appeared to be made up primarily of elemental sulfur and bacterial cells, with far less of the multi-cellular debris. This elemental sulfur is likely the byproduct of sulfide oxidation by the dominant *Epsilonproteobacteria, Sulfurovum*, and *Sulfurimonas*, which made up roughly half of all reads in the white floc and fluid samples. Isolates of *Sulfurovum* and *Sulfurimonas* from hydrothermal vent habitats can perform both the oxidation of various sulfur compounds to sulfate using the *sox* system and the oxidation of sulfide to elemental sulfur using sulfide-quinone reductase (*sqr*) (Inagaki et al., 2003; Nakagawa et al., 2007; Sikorski et al., 2010). These dominant genera likely require a very narrow range of redox conditions between warm, sulfide-rich diffuse flow and cold, oxygenated seawater as vent isolates of *Sulfurovum* and *Sulfurimonas* oxidize sulfide under microaerobic and mesophilic conditions (Inagaki et al., 2003; Nakagawa et al., 2007). While members of the genus *Arcobacter* may also perform sulfide oxidation with the production of elemental sulfur (Taylor and Wirsen, 1997) and have been detected as dominant members of other diffuse flow vents at Axial (Huber et al., 2007), here they constituted only a small fraction of all samples. *Sulfurovum* and *Sulfurimonas* have previously been identified as the most abundant and diverse groups in diffuse hydrothermal fluids in the Mid-Atlantic Ridge (Hügler et al., 2010) and in hydrothermal plumes at the East Pacific Rise (Sylvan et al., 2012). It is possible that the *Sulfurovum* and *Sulfurimonas* outcompete *Arcobacter* at snowblower vents during conditions of elevated hydrogen sulfide levels. A recent study of the microbial community in diffuse fluids from the Logatchev hydrothermal field found a significant correlation between sulfide enrichment and the proportions of *Sulfurovum* found (Perner et al., 2013), supporting the idea that *Sulfurovum* flourishes when sulfide levels are high.

It is not clear why only *Sulfurovum* was detected in clone libraries of *Epsilonprotoebacterial soxB* from white floc. The primer set used in this study was capable of recovering *Sulfurimonas*-like *soxB* from orange floc. It is possible that not all of the *Sulfurimonas* detected in these samples contain the same type of *soxB* that most *Epsilonproteobacteria* have and instead contain a *soxB* more similar to the distantly related *Arcobacter*-like *soxB* that does not amplify with these primers. Alternatively, deeper sequencing of the clone libraries may have revealed some *Sulfurimonas*-like *soxB*.

The snowblower fluid samples contained many of the same dominant taxa as the white floc, however, two groups clearly stood out as elevated in fluids, namely the *Gammaproteobacteria* SUP05 cluster and the *Methanosarcinales* GoM Arc 1 clade. Metagenomic and metatranscriptomic analyses have revealed that the uncultured SUP05 group also has the *sqr* gene for sulfide oxidation to elemental sulfur and that it expresses *sqr* as a member of hydrothermal vent plume communities (Anantharaman et al., 2013). SUP05 was also ubiquitous in fluid samples from the Main Endeavour Field on the Juan de Fuca Ridge, including background seawater, hydrothermal plumes, and hydrothermal diffuse flow (Anderson et al., 2012). Of these fluid types, SUP05 was most abundant in plume samples, making up almost a third of the total bacteria (Anderson et al., 2012). The *Methanosarcinales* were more abundant only in the Snow Globe fluid, but both fluid samples had higher proportions of total methanogens compared to the flocculent materials. This is likely a reflection that the strictly anaerobic methanogen populations are being flushed out from deeper subsurface layers in diffuse fluids, while the white floc is generated closer to the surface in microaerobic, mesophilic niches just below the seafloor.

Despite the apparent differences in circulation and chemistry between snowblower vents and more stable diffuse flow vents (Butterfield et al., 2004), our results show that the dominant organisms in snowblower fluids and white floc are similar to non-eruptive diffuse flow. Both the bacterial and archaeal communities in the white floc and the fluids were dominated by organisms previously found in diffuse flow at Axial Seamount, including, but not limited to, *Sulfurovum* and *Sulfurimonas* (Huber et al., 2003, 2007) and *Methanococcales* (Huber et al., 2002; Ver Eecke et al., 2012). The presence of mesophilic *Epsilonproteobacteria* and several strictly anaerobic, thermophilic archaeal groups such as *Methanococcus, Thermococcus*, and *Archaeoglobus* indicates that snowblower vents are seeded by subseafloor communities. During eruptive events, snowblower microbial communities form through two processes: the flushing of microbes from different thermal zones in the subsurface and the secondary bloom of microbes near the surface of the seafloor (Delaney, 1998). At Axial snowblower vents, the *Methanococci* and other thermophiles are likely present due to flushing, while the *Sulfurovum* and *Sulfurimonas* are likely residents of shallower subsurface layers and experiencing a secondary bloom just below the seafloor. Modeling of the biogenic production of white floc after the 1991 eruption at 9°N East Pacific Rise suggested that the source of material for snowblower vents may be a combination of microbial bloom and a flushing of accumulated floc within the seafloor (Crowell et al., 2008).

The peak activity of observed snowblower vents lasts from weeks to months, with microbial biomass diminishing as the sulfide levels decrease (Haymon et al., 1993). Snowblowers at Axial Seamount following the 1998 eruption were inactive 18 months post eruption (Butterfield et al., 2004). Though short-lived, snowblower vents provide episodic pulses of energy-rich fluids and chemolithoautotophic microbial communities to the surface of the seafloor and therefore impact ocean biogeochemistry, especially sulfur and carbon cycling. In particular, snowblower vents may be a significant source of biogenic elemental sulfur through sulfide oxidation by *Epsilonproteobacteria* such as *Sulfurovum, Sulfurimonas*, and *Arcobacter*, using either the Sox pathway or the sulfide-quinone reductase (*sqr*) gene. *Gammaproteobacteria*, such as sulfur-oxidizing members of the *Thiotrichales*, may also contribute to biogenic elemental sulfur production. Isolates of the *Gammaproteobacterial* genus *Thiomicrospira* from multiple vent systems in the Atlantic and Pacific are known to oxidize sulfur with the production of elemental sulfur (Wirsen et al., 1998) and the partial genomes of *Beggiatoa* filaments contain both *sox* and *sqr* genes (Mussmann et al., 2007). Members of the order *Thiotrichales* were detected at low levels in all of the fluid and white floc samples and were one of the most dominant groups in the orange floc. Finally, representatives of the *Gammaproteobacteria* clade SUP05 may also perform sulfide oxidation with *sox* and *sqr* genes (Anantharaman et al., 2013) both during eruptive events, as indicated by its presence in snowblower vents, and between eruptive events (Breier et al., 2012).

In summary, our observations of snowblower microbial communities substantiate the model of microbial responses to submarine eruptions described by Delaney (1998) which includes the transportation of subseafloor microbes out into the water column and a microbial population bloom in response to elevated levels of reduced compounds such as sulfide and iron. We saw clear differences between white and orange flocculent materials, with the white floc containing high levels of sulfur and the orange floc containing sheath-like structures similar to those in iron rich microbial mats. While quantitative PCR indicated that bacteria greatly outnumber archaea, the differences in both bacterial and archaeal community composition are informative to distinguish the sample types. We detected both *Epsilon*- and *Gammaproteobacteria* groups that are capable of sulfur oxidation in all microbial communities associated with snowblower vents. In addition, *Beta*- and *Gamma-proteobacteria* groups capable of iron oxidation were detected in the orange floc coating the seafloor around snowblower vents. These sulfur and iron oxidizing groups take advantage of the transient energy sources provided by deep-sea eruptions and are seeded from both subseafloor and bottom seawater communities. Future analyses will include metagenomic and metatranscriptomic work to determine the metabolic potential of the dominant and active members of snowblower communities.

## Conflict of interest statement

The authors declare that the research was conducted in the absence of any commercial or financial relationships that could be construed as a potential conflict of interest.

## Supplementary materials

Video S1 **Sample collection of white and orange flocculent materials at Axial Seamount, August 2011**.
